# Post-Bariatric Hypoglycemia: an Impaired Metabolic Response to a Meal

**DOI:** 10.1007/s11695-024-07309-y

**Published:** 2024-08-17

**Authors:** Ömrüm Aydin, Abraham S. Meijnikman, Patrick A. de Jonge, Karlijn van Stralen, Hanneke Börger, Kadriye Okur, Zainab Iqbal, Moritz V. Warmbrunn, Yair I. Z. Acherman, Sjoerd Bruin, Maaike Winkelmeijer, Alinda W. M. Schimmel, Jens J. Holst, Steen S. Poulsen, Fredrik Bäckhed, Max Nieuwdorp, Albert K. Groen, Victor E. A. Gerdes

**Affiliations:** 1 Department of Vascular Medicine, Amsterdam UMC – AMC, Amsterdam, the Netherlands; 2 Department of Scientific Research, Spaarne Gasthuis, Hoofddorp, the Netherlands; 3 Department of Bariatric Surgery, Spaarne Gasthuis, Hoofddorp, the Netherlands; 4 Cardiometabolic Research, Vrije Universiteit, Amsterdam, the Netherlands; 5 Novo Nordisk Foundation Center for Basic Metabolic Research, Copenhagen, Denmark; 6 Department of Biomedical Sciences, University of Copenhagen, Copenhagen, Denmark; 7 Department of Cardiovascular and Metabolic Research, Wallenberg Laboratory, Institute of Medicine, University of Gothenburg and Sahlgrenska University Hospital, Gothenburg, Sweden; 8 Department of Clinical Physiology, Region Västra Götaland, Sahlgrenska University Hospital, Gothenburg, Sweden; 9 Department of Internal Medicine, Spaarne Gasthuis, Hoofddorp, the Netherlands

**Keywords:** Bariatric surgery, Hypoglycemia, GLP-1, FGF-19, FGF-21, Gluconeogenesis

## Abstract

**Aims/Hypothesis:**

Post-bariatric hypoglycemia (PBH) is caused by postprandial hyperinsulinemia, due to anatomical alterations and changes in post-prandial metabolism after bariatric surgery. The mechanisms underlying the failing regulatory and compensatory systems are unclear. In this study, we investigated the differences in post-prandial hormones and metabolic profiles between patients with and without PBH.

**Methods:**

We performed a mixed meal test (MMT) in 63 subjects before and 1 year after Roux-en-Y gastric bypass (RYGB) surgery. Blood was withdrawn at 0, 10, 20, 30, 60, and 120 min after ingestion of a standardized meal. Glucose, insulin, GLP-1, FGF-19, and FGF-21 were measured and untargeted metabolomics analysis was performed on blood plasma to analyze which hormonal and metabolic systems were altered between patients with and without PBH.

**Results:**

Out of 63, a total of 21 subjects (33%) subjects developed PBH (glucose < 3.1 mmol/L) after surgery. Decreased glucose and increased insulin excursions during MMT were seen in PBH (*p* < 0.05). GLP-1, FGF-19, and FGF-21 were elevated after surgery (*p* < 0.001), but did not differ between PBH and non-PBH groups. We identified 20 metabolites possibly involved in carbohydrate metabolism which differed between the two groups, including increased carnitine and acylcholines in PBH.

**Conclusion:**

Overall, 33% of the subjects developed PBH 1 year after RYGB surgery. While GLP-1, FGF-19, and FGF-21 were similar in PBH and non-PBH patients, metabolomics analysis revealed changes in carnitine and acyclcholines that are possibly involved in energy metabolism, which may play a role in the occurrence of PBH.

**Graphical Abstract:**

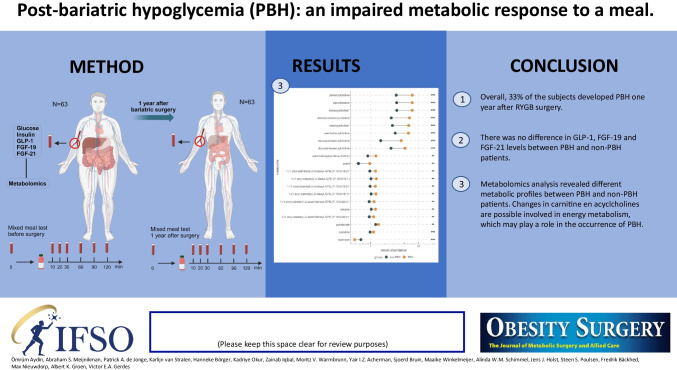

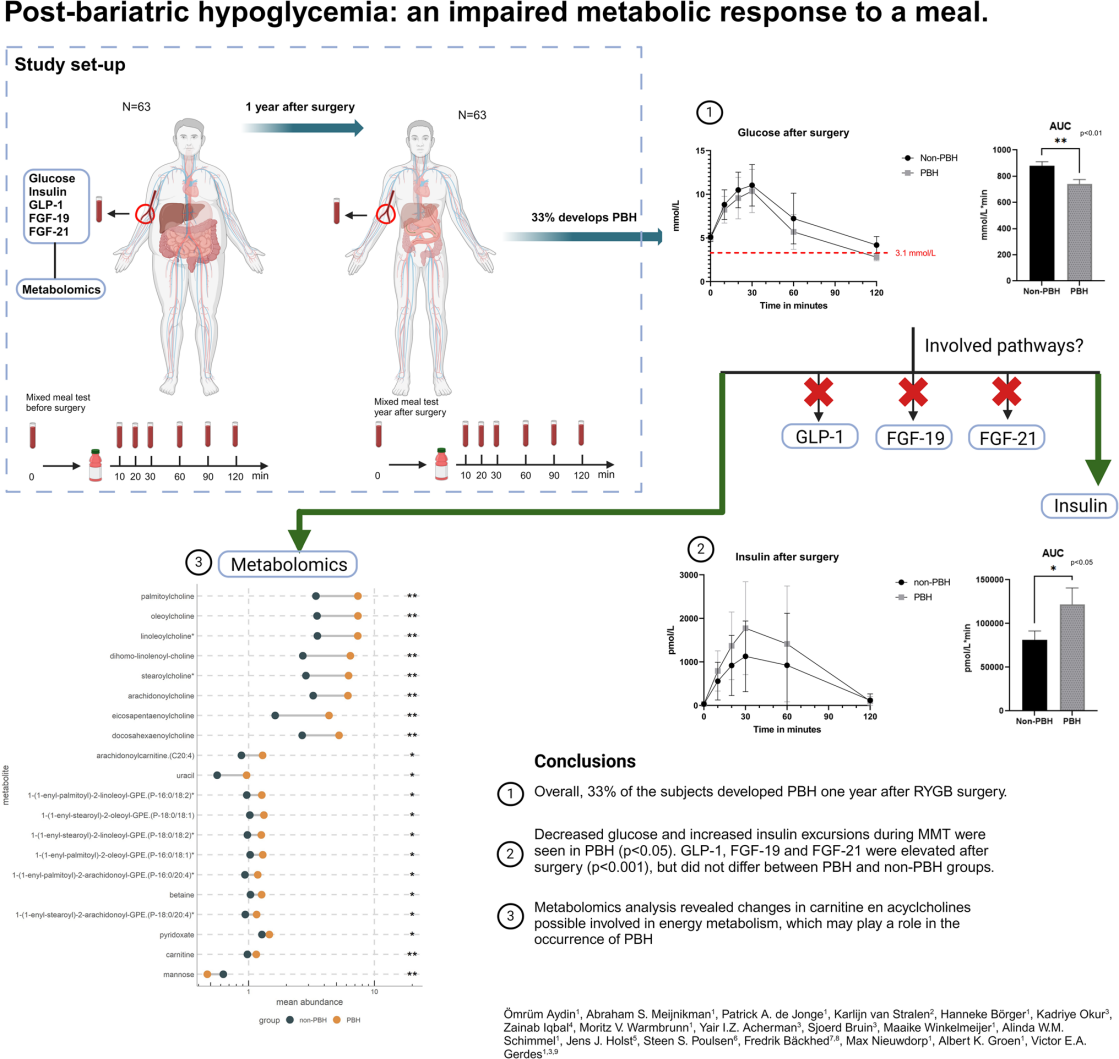

**Supplementary Information:**

The online version contains supplementary material available at 10.1007/s11695-024-07309-y.

## Introduction

Bariatric surgery is the most effective therapy for severe obesity. However, there is a considerable chance of long-term side effects after surgery. Recent research indicates that approximately one-third of individuals subjected to gastric bypass surgery develop post-bariatric hypoglycemia (PBH) within 1–4 years post-surgery [1–3]. PBH can be profoundly disabling, manifesting symptoms ranging from fatigue and headaches to severe neuroglycopenia, leading to loss of consciousness and/or seizures, sometimes necessitating hospitalization [4, 5]. The treatment consists of strict dietary advice and if necessary medication, which is not always effective. An understanding of the pathophysiology is imperative, not only for the development of effective treatments but also for early identification of patients at risk for PBH.

In recent years, considerable attention has been devoted to studying the post-prandial response after bariatric surgery [3, 6, 7]. This involves observing a rapid hyperglycemic peak after meal ingestion, accompanied by a hyperinsulinemic response within the first 30–60 min, followed by a steep hypoglycemic drop after 2 h of post-ingestion. Interestingly elevated levels of incretin hormones, particularly GLP-1, are described after surgery and hypothesized to be causal factors in PBH. [1, 8–10]

The elevated glucose and incretin hormone levels after a meal are likely attributed to the increased intensity of nutrient flow due to anatomical changes, potentially causing an overcompensation of insulin secretion and subsequent hypoglycemia. Changes in the dynamics of GLP-1, FGF-19, and FGF-20 after surgery have been proposed as causal factors in PBH [11, 12]. Studies also indicate the occurrence of gut hypertrophy, with increased numbers of enteroendocrine L cells per mm^2^, as a contributing factor to the elevated incretin response. [13–15]

Given the incomplete understanding of the pathophysiology, clinicians and researchers are experimenting with interventions that target various pathways. These interventions may reduce hypoglycemia symptoms by inducing gluconeogenesis, reducing insulin secretion, or mitigating the initial hyperglycemic peak, which mainly treats the symptom, but not the cause.

While previous research has provided insights into the post-prandial response, several questions remain unanswered. Despite GLP-1 elevation in all post-surgery patients, why does it lead to PBH in only one-third of cases? In a normal physiological state, the body has many counterregulatory mechanisms to prevent hypoglycemia. One of them is establishing a glucose threshold (around 5.0 mmol/L) [16], limiting insulin secretion and stimulating glucagon production; why is this threshold overridden after surgery? Additionally, the liver should theoretically compensate for hypoglycemia through gluconeogenesis; why is this feedback loop not effective enough? Interestingly, there is some evidence on the metabolic profiles after bariatric surgery suggesting differences in energy regulating mechanisms [17, 18]. However, this is based on one case report and a study with small sample size. Further research is needed to address these questions.

Our objective was to conduct a comprehensive investigation into the potential metabolic pathways involved in PBH, performing mixed meal tests before and after bariatric surgery in patients with severe obesity. Concentrations of the central hormones in glucose homeostasis as well as untargeted metabolomics in plasma from a bariatric surgery cohort.

## Methods

For this prospective study, we selected 63 subjects undergoing Roux-en-Y gastric bypass (RYGB) surgery. Patients participated in the longitudinal BARIA cohort study [19], and had completed mixed meal test before surgery as well as 1 year after surgery. In- and exclusion criteria are presented in supplementary Table 1. Study protocols were approved by the Ethical Review Board of the Academic Medical Center, Amsterdam, (approval code: NL55755.018.15). All procedures performed in this study were in accordance with the ethical standards of the institutional and/or national research committee and with the 1964 Helsinki declaration and its later amendments or comparable ethical standards. Informed consent was obtained from all individual participants included in the study.

### Mixed Meal Test and Data Collection

Before and 1 year after bariatric surgery (*T* = 0 and *T* = 1, respectively) a 2-h mixed meal test (MMT) was performed. Two servings of Nutridrink (Nutricia®) containing together 23.3 g of fat, 74.3 g of carbohydrates, and 24.0 g of protein were ingested. Blood was sampled via a peripheral venous catheter at 0, 10, 20, 30, 60, and 120 min from start of ingestion.

### Laboratory Investigations

All measurements were performed on plasma collected in lithium heparin tubes. Glucose was determined at every time point by the diagnostic laboratory in the University Medical Center Amsterdam (AUMC). Insulin was measured using the Immunometric assay, Luminescence, Atellica IM, Siemens at the laboratory of endocrinology AUMC. Plasma GLP1 levels were determined using ELISA (Mercodia) as previously described [20]. Plasma FGF-19 and FGF-21 were also measured by ELISA (R&D Systems). Untargeted metabolomics on plasma metabolites was performed by Metabolon (Durham, NC, USA) using ultra-high-performance liquid chromatography–coupled to tandem mass spectrometry (UPLC-MS/MS). A total of 858 annotated and 210 unannotated plasma metabolites were measured. Raw data were normalized to account for inter-day measurement differences. Then, data were rescaled so that the median level for each metabolite across all samples was equal to 1. Missing values, generally due to the metabolite levels falling below the limit of detection, were then imputed with half the minimum observed value for the respective metabolite across all samples.

### Immunohistochemistry of L Cells

The mucosal jejunal biopsies were taken 150 cm distally from the pylorus during surgery, before performing the jejuno-jejunostomy. The obtained biopsies were immediately fixated in formalin and embedded in paraffin. For immunohistochemistry, biopsies were cut about 5-µ thin and dewaxed through Tissue-Clear (Sakura Finetek. cat. No. 1466). Subsequently, antigen retrieval was performed in MBO for 15 min in 10 citric acid buffer (10 mM, pH 6). Next pre-incubation was performed in 2% BSA (wt/v) for 10 min followed by overnight incubation at 40 °C with the primary antibody: GLPa-1F5-6–2-2006, an “in house” mouse monoclonal, diluted 1:1500. For amplifying the immunoreactions, the sections were incubated for 40 min with biotinylated secondary antibody immunoglobulins, diluted at 1:200, followed by a preformed Avidin and Biotinylated horseradish peroxidase macromolecular complex (ABC) (Code nr. PK-4000 Vector Laboratories) as the third layer with a 30-min incubation time. After the second layer, endogenous peroxidase was blocked with 3% hydrogen peroxide. Finally, the reaction was developed by the addition of DAB (3.3–diaminobenzidine) (Code nr. SK-4100 Vector Laboratories) for 15 min. Counterstaining was performed with Mayer’s hematoxylin.

### L-Cell Count

The concentration and distribution of enteroendocrine L cells were evaluated on representative biopsy slide sections based on immunohistochemical staining. Digital images of biopsy slides were obtained using Image-Pro Plus version 9.1 with a 20 × objective. Secondly, the enteroendocrine cell density was measured by manual counting. A second observer performed a similar manual count; the mean count was used for the subsequent analysis. In total, 63 biopsy slide sections were produced and analyzed. The size of the epithelial area was obtained using a pre-specified grid and point-counting technique; the number of + points “hitting” a structure of interest was multiplied by the area per point yielding the total epithelial area. Next, the number of all immunopositive (stained) cells within the epithelial area was counted and divided by the size of the epithelial area.

### Statistical Analysis

Descriptive statistics were used for patient characteristics and results of laboratory measurements (GraphPad Prism 9.2.0). PBH was defined as a blood glucose level of ≤ 3.1 mmol/L after MMT. Insulin resistance was determined by calculating the homeostatic model assessment for insulin resistance (HOMA-IR) as follows: ((fasting glucose × fasting insulin)/22.5) after adjusting for units. The area under the curve (AUC) was calculated using the trapezoidal rule in Prism. Spearman’s rank was used for the correlation analyses as the current data did not meet the assumption of normal distribution and was neither linearly correlated to the outcome measure. For the same reason, Wilcoxon ranked test was used to study the differences between pre- and post-surgical values. To evaluate differences between patients with and without PBH, we performed either unpaired *T*-tests or Mann–Whitney *U* tests depending on the distribution of the data. Multiple logistic regression was performed to identify whether type 2 diabetes and/or type of surgery were confounding factors in the analysis. We also performed a sensitivity analysis on the glucose threshold of 3.1 mmol/L, using different thresholds between 2.7 and 3.4 mmol/L. A significance level of 5% was used; therefore, values with a *p*-value of < 0.05 were considered to be statistically significant.

Metabolomics statistical analyses and visualizations were performed in R (v. 3.6.2) using the tidyverse, ggpubr, and ggplot2 package. To test whether metabolites were significantly altered between the two patient groups, we calculated a non-parametric Wilcoxon ranked-sum test for each metabolite. *Q*-values were calculated using the Benjamin-Hochberg procedure [21]. Statistical tests were performed on metabolites after grouping them into their respective sub-pathways.

## Results

**Fig. 1:**
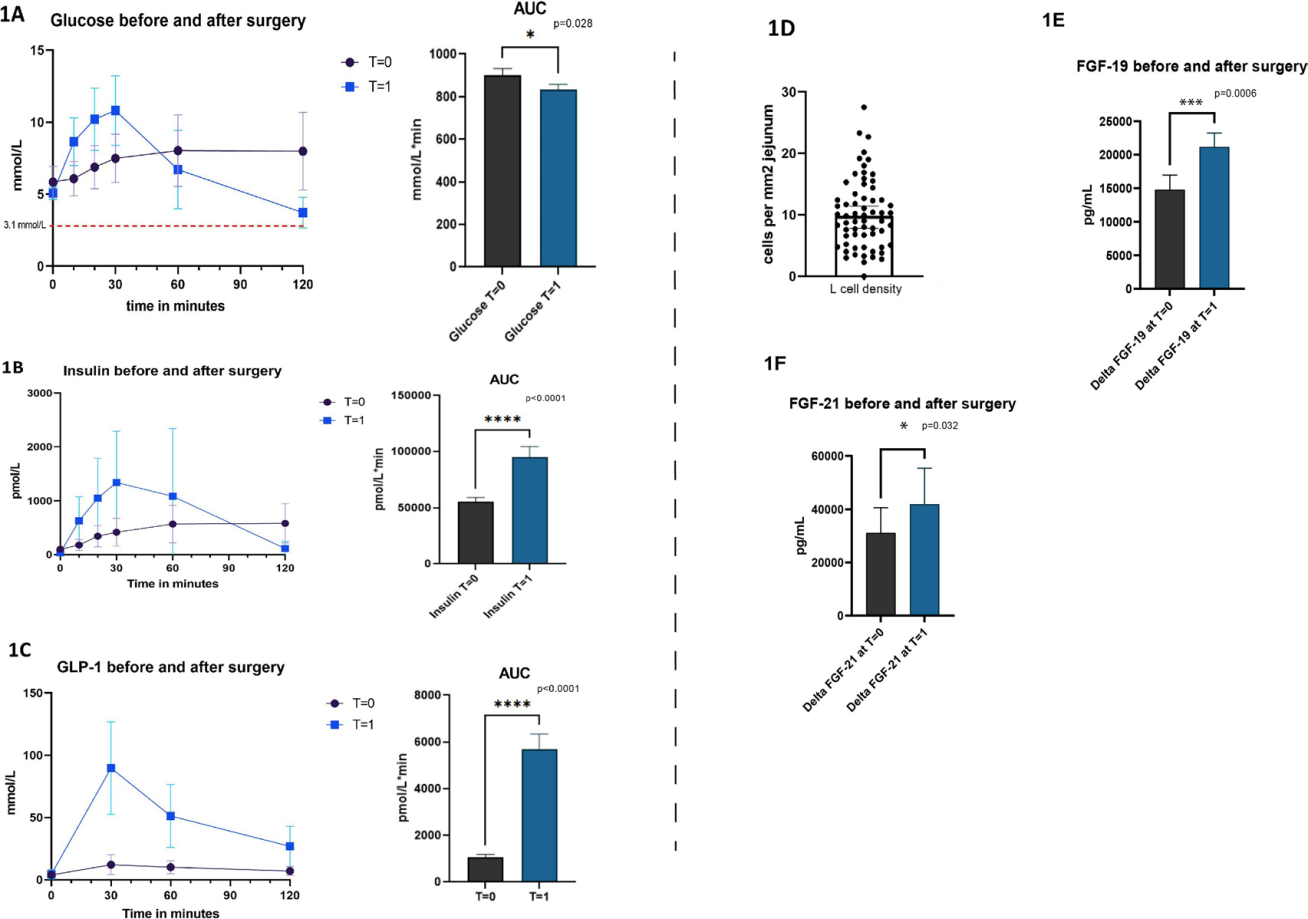
Hormonal dynamics of all subjects before (T=0) and after surgery (T=1) and L cell density. Representation of the mixed meal test curves with mean values and AUC before and after surgery. **A** Glucose,** B** insulin, and** C** GLP-1 excursions. Panel **D** represents L-cell density per mm^2^ of jejunal tissue. **E** Delta FGF-19. **F** Delta FGF-21. Error bars display 95%CI

**Fig. 2:**
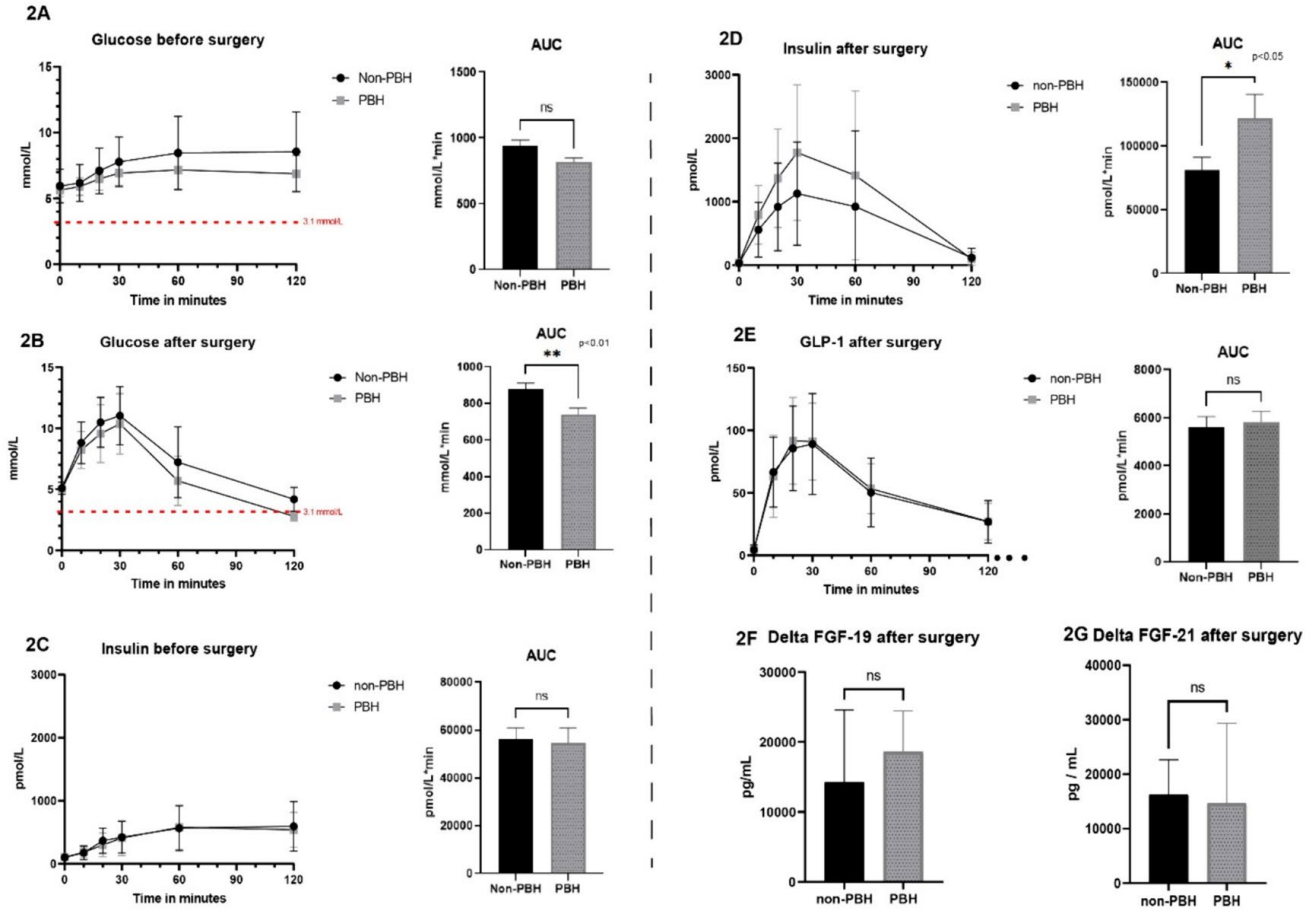
Hormonal dynamics after surgery between subjects with and without PBH. Representation of mixed meal test curves after surgery with mean values and AUC. **A&B **Glucose, **C&D **insulin, **E **GLP-1 curves after surgery, **F **AUC FGF-19 after surgery, and **G **AUC FGF-21 after surgery. Error bars represent standard error of the mean (SEM)

A total of 63 patients was included. The mean BMI was 40.4 kg/m^2^ ± 4.5 kg/m^2^ at *T* = 0, 46 subjects (73.5%) were female and 14 patients (22.2%) had T2D. The baseline characteristics are given in Table 1. At baseline and 1 year after bariatric surgery, the patients were subjected to an MMT. As shown in Fig. 1A, 1 year after surgery postprandial glucose excursions were significantly decreased (AUC baseline 900.5 ± 248.5 mmol/L*min versus AUC 1 year after surgery 833.2 ± 198.3 mmol/L*min, *p* = 0.028) and consisted of two phases; a steep postprandial glucose increase (phase 1) followed by a rapid decrease after 30 min (phase 2). Of the total group, 21 patients (33.3%) experienced a glucose level lower than 3.1 mmol/L during the MMT and were classified as PBH, the other 42 patients were classified as non-PBH. Figure 2A and B depicts the postprandial glucose excursions and AUC before surgery and after 1 year for both the PBH group and non-PBH group. An overview of the correlations between the hormones after surgery in the PBH and the non-PBH group is presented in supplementary Table 2. The AUC of the glucose excursion in the PBH group was significantly lower at 1 year (*p* < 0.01), whereas before surgery there was no significant difference between both groups. Regarding insulin, Fig. 2B and C shows a clear difference in the insulin excursion and AUC of the PBH group 1 year after surgery (*p* < 0.05), whereas before surgery the AUCs were similar. Insulin resistance measured as HOMA-IR did not differ between the groups.

**Table 1:** Baseline characteristics of the study population

Study population	*N* = 63
Age (years)	47.14 ± *9.8 SD*
Sex - Female (%) - Male (%)	46 (73.0%) 17 (27.0%)
Surgery type: - RYGB (%) - Omega-loop gastric bypass (%)	59 (93.7%) 4 (6.3%)
Weight before surgery (kg)	121.7 ± *15.6 SD*
Body mass index before surgery (kg/m^2^)	40.64 ± *4.4 SD*
Diabetes mellitus type II	13 (20.6%)
Hypertension	19 (30.2%)
Medication use	41 (65.1%)

Continuous data presented as mean ± SD, categorical data presented as *N* (%)

*RYGB* Roux-en-Y gastric bypass

To investigate a possible role of L-cell activity we assessed L-cell density in jejunal biopsies. No difference between PBH and non-PBH groups was found (supplementary Fig. 1). In line with this observation, GLP-1 excursions during the MMT were completely identical between PBH and non PBH (Fig. 2E).

To investigate whether the cutoff of glucose at 3.1 mmol/L impacts the data analysis, we performed a sensitivity analysis using different cut-offs for glucose between 2.7 and 3.4 mmol/L. A different threshold however did not significantly affect the AUC comparisons as shown in Fig. 2.

Fibroblast growth factor 19 and 21 have been reported to play an important role in glucose homeostasis. We therefore also measured the concentration of these hormones before and after bariatric surgery and both were significantly increased after surgery (Fig. 1E and F). Yet, as depicted in Fig. 2F and G, no differences in delta FGF-19 or delta FGF-21 were seen between PBH and non-PBH groups. Supplemental Fig. 2 shows the correlations between the abovementioned hormones after surgery.

### Plasma Metabolites in Relation to Post-Bariatric Hypoglycemia (PBH)

**Fig. 3:**
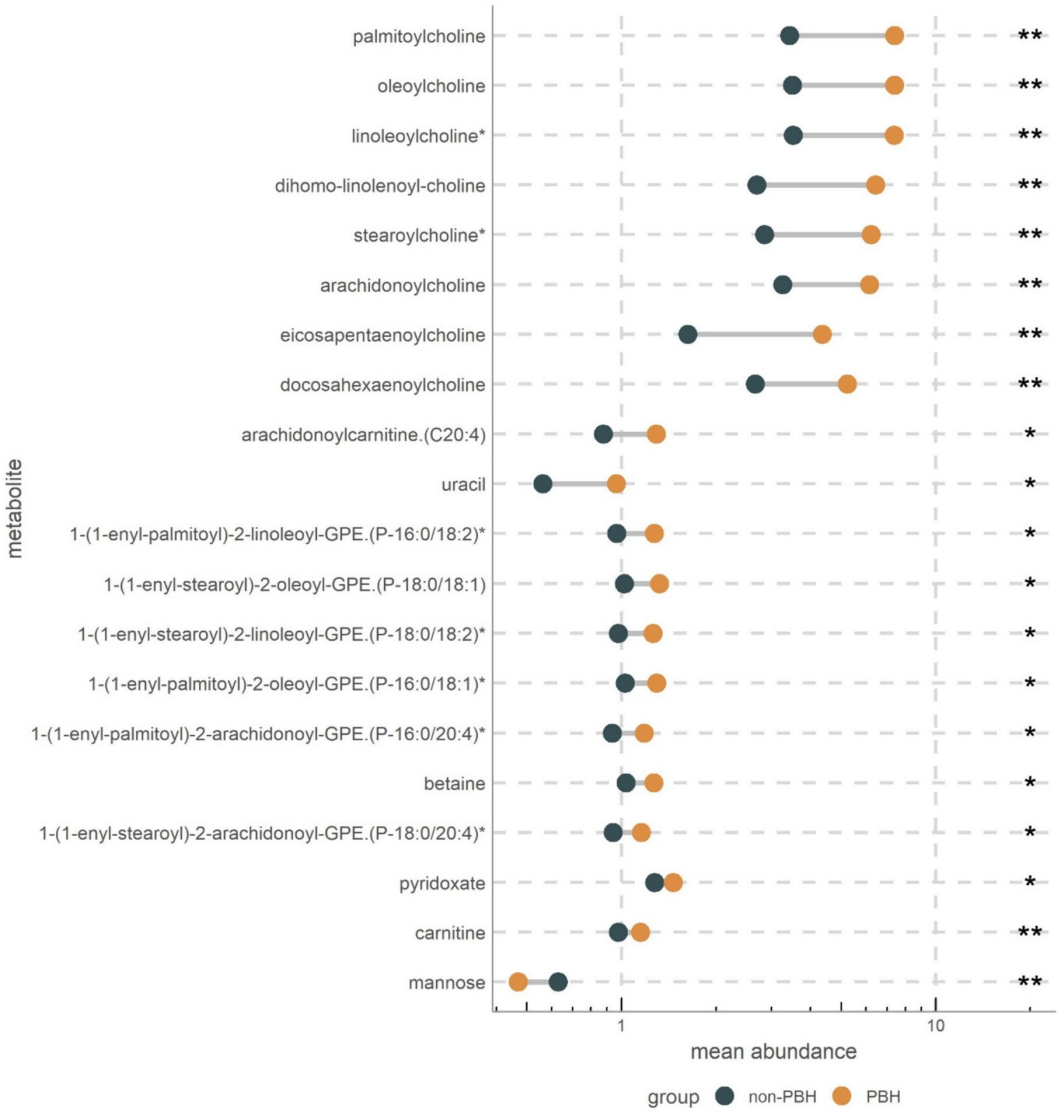
Metabolomics in PBH. Cleveland plot of the 20 significantly different metabolites between PBH and non-PBH group. Dots represent mean abundance in the two groups; with mean abundance in log scale on the *X*-axis. *Y*-axis represents the metabolites. Significance is reported as asterisk (*p < 0.05 and **p < 0.01) after correction for multiple testing according to Benjamini–Hochberg method. Additional information on involved pathways and exact *q*-values are presented in supplementary Table 3

Thus, except for insulin, the above-measured hormones did not directly account for the difference in glucose levels after 2 h. Since plasma metabolites mirror other aspects of energy metabolism, we performed plasma metabolomics taken at 120 min of MMT for differences between the PBH and non-PBH groups 1 year after surgery. Postprandial metabolomics (120 min) analysis identified 20 metabolites as being significantly different in PBH even after correction for multiple testing. Increased carnitine, acylcholines, and plasmalogens were observed in the PBH group (Fig. 3 and supplemental Table 3). MMT induced an increase in carnitine (*q* = 0.009) in the PBH group with a concomitant increase in all acylcholines present in the metabolomics panel including linoleoylcholine (*p* = 0.003), oleoylcholine (*q* = 0.003), palmitoylcholine (*q* = 0.003), arachidonoylcholine (*q* = 0.004), eicosapentaenoylcholine (*q* = 0.004), stearoylcholine (*q* = 0.004), dihomo-linolenoyl-choline (*q* = 0.004), and docosahexaenoylcholine (*q* = 0.006). Furthermore, plasma metabolites such as mannose (*q* = 0.003), pyridoxate (*q* = 0.02), betaine (*q* = 0.03), uracil (*q* = 0.031), and arachidonoylcarnitine (*q* = 0.046) were also significantly different between the two groups (Fig. 3 and supplemental Table 3).

## Discussion

In our search for the culprits causing PBH, we measured an extended array of factors that have been suggested to play a role in induction of hypoglycemia after bariatric surgery. We could not confirm a direct role for GLP-1, which was reported in other studies [6, 10]. However, by carrying out non-targeted plasma metabolomics, we did obtain an indication for an interesting novel pathway. We observed increased levels of acylcholines that may signal inhibition of mitochondrial fatty acid beta-oxidation inducing stimulation of glycolysis thus lowering plasma glucose levels. Although our findings are preliminary and need reconfirmation in larger cohorts, we propose that acylcholines might be players in the development of PBH.

The anatomical changes induced by RYGB surgery are known to cause an increased glucose uptake in the first 30 min after a meal [22]. We confirmed a pronounced insulin and GLP-1 response 1 year after surgery, but for the latter there was no difference between the PBH and non-PBH groups. Insulin production did significantly differ between the two groups (*p* < 0.05), but the differences are mainly in the first 30 min after ingestion of a meal. Interestingly, at 120 min after initiating the MMT, insulin levels were identical in PBH and non-PBH groups. In addition, patients with and without PBH were characterized by similar GLP1, FGF-19, and FGF-21 plasma levels and the same amount of enteroendocrine L-cell density in the jejunum. Also, our observed prevalence of PBH of 33.3% is in line with other reports [2]. Previous studies suggested this to be a result of pancreatic beta-cell hyperplasia and hypertrophy, possibly as a result of the increased GLP-1 levels [4, 23]. However, although our results indicate that GLP1 contributes to the changed pattern after surgery, this incretin does not seem to explain the exaggerated response in our patients with PBH [24]. Also, post-mortem analysis did not show any pancreatic beta-cell hyperplasia after bariatric surgery [25]. Finally, although it was suggested that insulin resistance might be protective against PBH [26], we did not observe differences in HOMA-IR between the PBH and non-PBH groups.

In line with previous studies, we also confirmed that stimulated GLP-1 release was increased after surgery [9]. In this regard, it has been suggested that proliferation of jejunal enteroendocrine L cells could contribute to this response [27]. However, in our study, L-cell density at time of surgery did not show any correlation with GLP-1 or PBH.

Other researchers reported a possible non-insulin dependent role for FGF-19 in developing PBH, although the mechanism was unclear [12]. In our study, both FGF-19 and FGF-21 were significantly increased after bariatric surgery. FGF-19 did not correlate with insulin nor significantly with glucose and GLP-1 (supplemental Fig. 2). In this study, we also studied FGF-21 as a possible influencing factor in PBH. FGF-21 is known for improving insulin sensitivity and lowering blood glucose in mainly animal models [28]. FGF-21 did not differ between PBH and non-PBH patients.

To investigate a possible metabolic signature in the PBH group, we performed untargeted metabolomics. Several interesting metabolites surfaced in the analysis. We observed increased carnitine and in particular acylcholine levels in patients with PBH. Carnitine is a hydrophilic quaternary amine and is essential for beta-oxidation by transporting long-chain fatty acids over the inner mitochondrial membrane [29]. Carnitine accumulation may point to inhibition of carnitine palmitoyltransferase-1 (CPT-1) in the mitochondrial membrane. It is well known that insulin stimulates acetyl-CoA carboxylase leading to an increase in malonyl-CoA, the bonafide inhibitor of CPT-1. A decreased beta-oxidation will induce accumulation of long-chain fatty acyl-CoA. To salvage CoA, the cell may choose to transfer a choline moiety to the long-fatty acid, thus producing acylcholines. The metabolomics output contained linoleoylcholine, oleoylcholine, palmitoylcholine, arachidonoylcholine, eicosapentaenoylcholine, stearoylcholine, dihomo-linolenoyl-choline, and docosahexaenoylcholine. All were increased in the PBH group, suggesting that indeed this mechanism may be operative. Interestingly, next to acylcholines, plasmalogens were also increased. Acyl-CoA’s play a pivotal role in synthesis of plasmalogens [30]. We speculate that the increased levels of plasmalogens are induced by enhanced fatty acyl-CoA.

Could such a metabolic diversion explain the induction of PBH. The only clear difference between PBH and non-PBH was the increased insulin excursion. In combination with the restored insulin sensitivity after RYGB surgery, the increased insulin in PBH may just have tipped the balance and induced extra inhibition of beta oxidation, and as consequence increased glycolysis leading to decreased plasma glucose. Note that acylcholines have been suggested to interfere with acetylcholine signaling. It has been shown that pancreatic beta cells express the M3-muscarinic acetylcholine receptor. Mice in which the receptor was disrupted in the beta cell only show altered insulin signaling [31]. Acylcholines may interfere with this effect. Clearly these hypotheses require further investigation.

It is interesting to compare the metabolomic pattern in PBH with that observed for T2DM. In patients with diabetes, or insulin resistance, it is well known that fatty acyl-carnitines accumulate, indicating that mitochondria cannot cope with high influx of fatty acids in obese patients. This sequence of events is exactly opposite to what occurs in the PBH patients. Hence, the patients with T2D should show a decreased level of acylcholines and this was indeed observed [32, 33].

There are several limitations to our study such as the use of only two time points (0 and 120 min) for FGF-19 and FGF-21. Measuring the entire curve might have provided more insight in the interaction of these hormones with glucose. Second, we did not have repeated biopsies to investigate possible increased L-cell density after surgery [13, 15]. Finally, metabolomics data provided insight in relative concentrations, not absolute values of metabolites.

Collectively, our data showed that PBH might be caused by an impaired response to compensate for hypoglycemia by increasing gluconeogenesis. Carnitine accumulation, together with increased acylcholines, suggests impaired beta-oxidation possibly stimulating glycolysis in the PBH group. This novel insight may suggest new opportunities for therapeutic interventions against PHB.

## Supplementary Information

Below is the link to the electronic supplementary material.

Supplementary file1 (DOCX 111 KB)

## Data Availability

The original data are available on request from the authors.

## Abbreviations

*MMT*: Mixed meal test
*PBH*: Post-bariatric hypoglycemia
*RYGB*: Roux-en-Y Gastric Bypass
*GLP-1*: Glucagon-like Peptide 1
*FGF-19*: Fibroblast growth factor 19
*FGF-21*: Fibroblast growth factor 21
