# Supplementary material for: Post-Bariatric Hypoglycemia: an Impaired Metabolic Response to a Meal
Source: Obes Surg. 2024 Aug 17;34(10):3796–806. doi: 10.1007/s11695-024-07309-y (PMC11481667; doi:10.1007/s11695-024-07309-y)
Supplement: Supplementary file 1 — Supplementary file1 (DOCX 111 KB) [file 11695_2024_7309_MOESM1_ESM.docx]

**Supplementary material**

**Supplemental table 1:**

**Inclusion and exclusion criteria BARIA Study**

*Inclusion criteria:*

- Male and female subjects eligible for bariatric surgery and recruited from an experienced bariatric surgery clinic.
- Body Mass Index >35 kg/m^2^ with an obesity-related co-morbidity; or BMI >40 kg/m^2^ with / or without an obesity related co-morbidity
- Age between 18 and 65 years
- Ability to provide informed consent

*Exclusion criteria:*

- Previous bariatric surgery
- Primary lipid disorder
- Known genetic basis for insulin resistance or glucose tolerance
- Psychiatric conditions
- Coagulation disorders (patient reported or prolonged prothrombin time or activated partial thromboplastin time).
- Uncontrolled hypertension (blood pressure >150/95 mmHg)
- Renal insufficiency (creatinine > 150 µmol/L)
- Excessive alcohol intake (>14 units/week, patient reported)
- Pregnancy or breastfeeding

**Supplementary figure 1: L cell density in jejunum biopsies**

Medians and distribution of L cell density at time of surgery in the non-PBH and PBH group are depicted. Non-PBH has a median of 8.3 (IQR 4.8-12.3) and PBH 10.1 (IQR 8.2-16.20), which is not significantly different. Error bars represent 95% CI.

|  | **Hypo** | **Non hypo** | **Significance** |
| --- | --- | --- | --- |
| **N** | 21 | 42 |  |
| **Age (in years)** | 49.24 ± 7.5 | 46.1 ± 10.1 | ns |
| **Female** | 14 (66.7%) | 32 (76.2%) | ns |
| **Weight (kg) before surgery** | 124.0 ± 11.8 | 120.6 ± 17.2 | ns |
| **BMI (kg/m2) before surgery** | 40.0 ± 3.1 | 41.0 ± 4.9 | ns |
| **%T2D before surgery** | 4 (19%) | 9 (21.4%) | ns |
| **BMI (kg/m2) after surgery** | 28.4 ± 3.3 | 29.0 ± 5.1 | ns |
| **Weight (kg) after surgery** | 88.6 ± 12.1 | 85.2 ± 16.2 | ns |
| **Waist circumference after surgery** | 97.7 ± 11.2 | 97.3 ± 12.5 | ns |
|  |  |  |  |
| **Laboratory resuls after surgery:** |  |  |  |
| Hemoglobulin (mmol/L) | 8.4 ± 0.6 | 8.4 ± 0.9 | ns |
| Trombocytes (*10^^9^/L) | 238.0 ± 63.9 | 247.4 ± 49.1 | ns |
| Leukocytes (*10^^9^/L) | 5.4 ± 1.3 | 6.2 ± 1.9 | ns |
| CRP (mg/L) | 1.2 ± 1.2 | 1.5 ± 1.8 | ns |
| TSH (mIU/L) | 2.0 ± 1.7 | 1.7 ± 0.9 | ns |
| HbA1c (%) | 5.4 ± 0.2 | 5.4 ± 0.4 | ns |
|  |  |  |  |
| **HOMA-IR before surgery** | 3.3 (2.0-4.6) | 2.8 (2.0-4.1) | ns |
| **HOMA-IR after surgery** | 0.9 (0.7-1.4) | 1.0 (0.7-1.4) | ns |
|  |  |  |  |
| **AUC glucose (mmol/L x 120 min) after surgery** | 741.7 (SEM 33.2) | 878.9 (SEM 31.5) | p<0.01 |
| **AUC insulin (pmol/L x 120 min) after surgery** | 122002 (SEM 18,776) | 81565 (SEM 9,992) | P<0.05 |
| **AUC GLP-1 (pmol/L x 120 min) after surgery** | 5840 (SEM 428.6) | 5636 (SEM 426.3) | ns |
|  |  |  |  |
| **Delta FGF-19 (pg/mL) after surgery** | 23823 (SEM 4886) | 19833 (SEM 1962) | ns |
| **Delta FGF-21 (pg/mL) after surgery** | 60745 (SEM 37594) | 32494 (SEM 7774) | ns |

**Supplemental Table 2. Patients with PBH and patients without PBH: characteristics and measurements**

An overview of the characteristics between the PBH (blood glucose <3.1 mmol/L) and non-PBH group. Values are presented as mean with either ±SD or SEM.


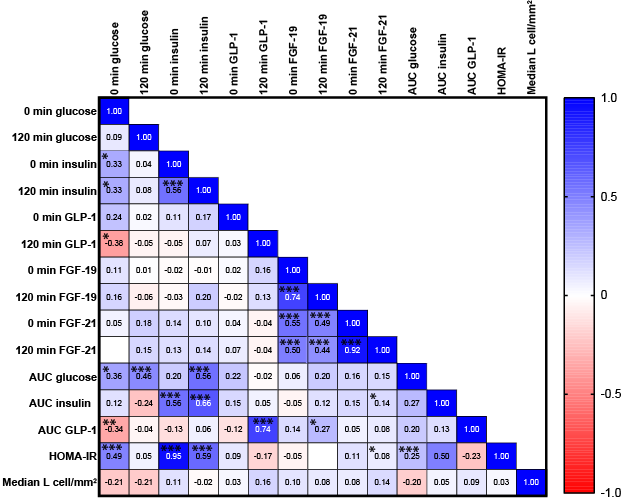


**Supplementary figure 2: Correlations between glucose, insulin, GLP-1, FGF-19 and FGF-21 after surgery.**

Correlation matrix between fasted (0 minutes), postprandial (120 minutes) and AUC’s of glucose, insulin, GLP-1, FGF-19 and FGF-21 and HOMA-IR and L cell density all after surgery. The direction of the correlation coefficient is color coded as seen in the legend; the correlation coefficient itself is noted in each box. Statistical significance is marked using asterisk: * for p<0.05, ** for p<0.01 and *** for p<0.001.

|  | **PATHWAY** | **Metabolite** | **q-value** |  |
| --- | --- | --- | --- | --- |
| 1 | Fatty Acid Metabolism (Acyl Choline) | linoleoylcholine | 0,0031 |  |
| 2 | Fatty Acid Metabolism (Acyl Choline) | oleoylcholine | 0,0031 |  |
| 3 | Fatty Acid Metabolism (Acyl Choline) | palmitoylcholine | 0,0031 |  |
| 4 | Fructose, Mannose and Galactose Metabolism | mannose | 0,0033 |  |
| 5 | Fatty Acid Metabolism (Acyl Choline) | arachidonoylcholine | 0,0040 |  |
| 6 | Fatty Acid Metabolism (Acyl Choline) | eicosapentaenoylcholine | 0,0040 |  |
| 7 | Fatty Acid Metabolism (Acyl Choline) | stearoylcholine | 0,0040 |  |
| 8 | Fatty Acid Metabolism (Acyl Choline) | dihomo-linolenoyl-choline | 0,0041 |  |
| 9 | Fatty Acid Metabolism (Acyl Choline) | docosahexaenoylcholine | 0,0056 |  |
| 10 | Carnitine Metabolism | carnitine | 0,0091 |  |
| 11 | Vitamin B6 Metabolism | pyridoxate | 0,0181 |  |
| 12 | Plasmalogen | 1-(1-enyl-palmitoyl)-2-arachidonoyl-GPC_(P-16:0/20:4)* | 0,0231 |  |
| 13 | Plasmalogen | 1-(1-enyl-palmitoyl)-2-linoleoyl-GPC_(P-16:0/18:2)* | 0,0231 |  |
| 14 | Plasmalogen | 1-(1-enyl-palmitoyl)-2-oleoyl-GPC_(P-16:0/18:1)* | 0,0231 |  |
| 15 | Plasmalogen | 1-(1-enyl-stearoyl)-2-oleoyl-GPE_(P-18:0/18:1) | 0,0231 |  |
| 16 | Glycine, Serine and Threonine Metabolism | betaine | 0,0301 |  |
| 17 | Pyrimidine Metabolism, Uracil containing | uracil | 0,0318 |  |
| 18 | Plasmalogen | 1-(1-enyl-stearoyl)-2-arachidonoyl-GPE_(P-18:0/20:4)* | 0,0348 |  |
| 19 | Plasmalogen | 1-(1-enyl-stearoyl)-2-linoleoyl-GPE_(P-18:0/18:2)* | 0,0348 |  |
| 20 | Fatty Acid Metabolism (Acyl Carnitine, Polyunsaturated) | arachidonoylcarnitine_(C20:4) | 0,0456 |  |
|  |  |  |  |  |

**Supplemental table 3: Overview of metabolomics pathways involved in PBH**

An overview of all the significantly different metabolites in PBH versus non-PBH together with their involved metabolic pathway. Significance is reported as q-value after correction for multiple testing according to Benjamini-Hochberg method.
